# Global, regional, and national burden of appendicitis among children and adolescents from 1990 to 2021 and projection to 2040: a cross-sectional study

**DOI:** 10.1097/JS9.0000000000003215

**Published:** 2025-10-13

**Authors:** Ying Huang, Dong Peng, Hongbo Huang, Tingting Wei, Chunyan Luo, Jiaying Li, Aijie Zhang, Ze Zhang, Zheng Gong, Zhiqi Hu, Yichen Wang, Guosheng Ren, Yunhai Li, Fan Li

**Affiliations:** a Department of Breast and Thyroid Surgery, The First Affiliated Hospital of Chongqing Medical University, Chongqing, China; b Department of General Surgery, The First Affiliated Hospital of Chongqing Medical University, Chongqing, China; c Department of Gastrointestinal Surgery, The First Affiliated Hospital of Chongqing Medical University, Chongqing, China; d Department of Gastroenterology, The Second Affiliated Hospital of Chongqing Medical University, Chongqing, China; e Health Management Center, University-Town Hospital Affiliated to Chongqing Medical University, Chongqing, China

**Keywords:** appendicitis, children and adolescents, global burden, Socio-demographic Index

## Abstract

**Background and importance:**

Appendicitis is a common surgical emergency in children and adolescents, yet its global epidemiological trends and burden remain understudied. This study aims to analyze the global burden of appendicitis in children aged 0–19 years from 1990 to 2021.

**Methods:**

We extracted data from the Global Burden of Disease 2021 study to analyze the incidence, prevalence, mortality, and disability-adjusted life-years (DALYs) attributable to appendicitis. Age-standardized rates and trends were stratified by sex, age group, region, and Socio-demographic Index (SDI).

**Results:**

In 2021, there were 4.53 million new cases of appendicitis and 360 249 DALYs among children and adolescents globally. The highest age-standardized incidence rate (270.38 [95% uncertainty interval {UI}, 180.93–386.54]) was observed in high-SDI regions, while the highest mortality rate (0.23 [95% UI, 0.14–0.35]) occurred in low-SDI regions. From 1990 to 2021, global age-standardized incidence and prevalence rates decreased slightly, while mortality and DALYs declined substantially. However, the age-standardized incidence and prevalence rates began increasing after 2015 and are projected to rise by over 21% by 2040. Frontier analysis highlighted strong performance in Somalia and Niger among low-SDI countries, whereas the United States Virgin Islands, a high-SDI region, exhibited considerable potential for improvement.

**Conclusions:**

The burden of appendicitis among children and adolescents remains significant, with pronounced inequalities across SDI levels. Addressing these inequities requires targeted interventions, such as improving surgical infrastructure in low-SDI regions and optimizing resource utilization in high-SDI regions. These findings provide a foundation for evidence-based policies to ensure equitable care for this vulnerable population.

## Introduction

Appendicitis, a leading acute abdominal condition globally, exhibits significant burden variations across populations and regions due to demographic and healthcare disparities^[1,2]^. Although predominantly affecting children and adolescents aged 10–20 years (accounting for >95% of acute abdomen cases), it is crucial to emphasize its potential severity in adult and elderly populations. Patients aged 80 years or older are at significantly higher risk of complications, with more severe clinical progression leading to mortality rates approximately 10-fold higher than those observed in adult populations. Furthermore, computed tomography (CT) scanning is mandatory for differential diagnosis in these cases^[1,3]^. Notably, in pediatric populations, acute appendicitis may conceal underlying pathologies such as appendiceal neuroendocrine neoplasms, with a significant proportion being diagnosed incidentally during postoperative histopathological examination^[4]^. Despite diagnostic and treatment advancements, appendicitis remains a top cause of pediatric emergency visits and hospitalizations^[5]^. At this critical developmental stage, the disease can cause severe complications, including postoperative issues, psychological impacts, and increased healthcare costs^[6,7]^. Despite its clinical significance and recent studies highlighting global increases in age-standardized incidence rates of appendicitis in nearly half of regions, most epidemiological research has primarily focused on the general population, with limited emphasis on children and adolescents, a group uniquely vulnerable due to their physiological and developmental characteristics^[1,8]^. A comprehensive evaluation of the epidemiological trends and regional disparities in appendicitis among children and adolescents is essential for targeted prevention strategies and resource allocation.

To address this knowledge gap, this study utilized data from the Global Burden of Disease Study (GBD) 2021 to systematically analyze the incidence, prevalence, deaths, and disability-adjusted life years (DALYs) attributable to appendicitis among individuals under 20 years of age from 1990 to 2021. The analysis encompasses temporal trends across age groups, sexes, and geographic regions, providing a detailed and comparable assessment of appendicitis burden in children and adolescents. By elucidating these trends, our findings aim to inform global health strategies, support evidence-based policy making, and guide the allocation of healthcare resources to improve the prevention and management of appendicitis in this vulnerable population.

## Methods

### Data sources

Data on appendicitis from 1990 to 2021 were sourced from the GBD 2021 database, which was accessed using the Global Health Data Exchange online query tool (http://ghdx.healthdata.org/gbd-results-tool). The GBD study systematically collects and models data from 204 countries and territories using over 87 000 data sources, including vital registration systems, verbal autopsies, hospital inpatient discharges, and linked insurance claims (e.g. the US Truven database and national programs in Poland and Taiwan). Appendicitis cases were identified using International Classification of Diseases, Tenth Revision (ICD-10) codes K35-K37.9 (acute/unspecified/other appendicitis) and K38.3-K38.9 (fistula/unspecified appendix diseases)^[2,9]^. Mortality estimates were generated using the Cause of Death Ensemble model, which integrates death records with sociodemographic covariates including the Socio-demographic Index (SDI), Healthcare Access and Quality Index, education, income, and dietary factors after redistributing garbage codes and applying Bayesian noise reduction, while incidence and prevalence were modeled through DisMod-MR 2.1, a Bayesian meta-regression tool that synthesizes claims data (defining incident cases as ≥1 ICD-coded encounter with 28-day readmission exclusion) and hospital discharge records (standardized for stays ≥24 hours), with inputs adjusted via meta-regression-derived correction factors for non-primary diagnoses and outpatient cases. Years lived with disability (YLDs) incorporated a disability weight of 0.32 (95% uncertainty interval [UI]: 0.22–0.44), years of life lost (YLLs) used reference life expectancy, and DALYs were calculated by summing YLLs and YLDs, with 95% UIs estimated through 1000 posterior draws to propagate errors from data sampling, model parameters, and covariate estimation. Study reliability was enhanced by excluding outliers (>2 median absolute deviations from global incidence medians), adjusting for commercial insurance biases, and adhering to Guidelines for Accurate and Transparent Health Estimates Reporting guidelines. This analysis focuses on appendicitis burden (incidence, prevalence, mortality, and DALYs) in the first two decades of life (0–19 years), categorized into four age subgroups: <5, 5–9, 10–14, and 15–19 years. We also used the SDI, a composite index reflecting a country or region’s sociodemographic status through income, education, and fertility rates^[10]^. In studying the correlation between SDI and the burden of appendicitis, we also included mortality-to-incidence ratios (MIR) as part of the analysis^[11]^.

**HIGHLIGHTS**

- Global burden and trends: The study reveals that in 2021, there were 4.53 million new cases and 360 249 disability-adjusted life-years (DALYs) due to appendicitis among children and adolescents globally, with significant disparities across Socio-demographic Index (SDI) levels. High-SDI regions had the highest incidence rates, while low-SDI regions faced the highest mortality rates.
- Rising incidence projections: Despite a slight decline from 1990 to 2021, age-standardized incidence and prevalence rates of appendicitis began increasing after 2015 and are projected to rise by over 21% by 2040, highlighting a growing public health challenge.
- Socioeconomic disparities: The study identifies pronounced inequalities, with high-SDI regions experiencing higher incidence and prevalence due to advanced diagnostics and lifestyle factors, whereas low-SDI regions suffer from higher mortality and DALYs due to limited healthcare access and delayed treatment.
- Frontier analysis insights: Countries like Somalia and Niger (low-SDI) demonstrate strong performance in managing appendicitis burden despite limited resources, while high-SDI regions such as the United States Virgin Islands show significant potential for improvement in reducing mortality and DALYs.
- Call for targeted interventions: The findings underscore the need for tailored strategies, including improving surgical infrastructure in low-SDI regions and optimizing resource utilization in high-SDI regions, to address inequities and reduce the global burden of appendicitis in children and adolescents.

### Statistical analysis

The burden of appendicitis was expressed as numerical counts and age-standardized rates per 100 000 population, stratified by sex, age, year, region, country, and SDI. Joinpoint regression was used to assess the annual average percentage change (AAPC) trends from 1990 to 2021. The AAPC offers a summary of these trends over a defined period by calculating a weighted average of the annual percent changes (APCs).

We employed the Bayesian age-period-cohort (BAPC) model, implemented within the integrated nested Laplace approximation (INLA) framework, to predict age-standardized rates of appendicitis burden (including incidence, prevalence, mortality, and DALYs) from 2022 to 2040^[12,13]^. These projections were based on global population estimates for 2017–2100. Compared with conventional methods, this approach provides superior computational efficiency and reduced error rates. By integrating the BAPC model with the INLA framework, we obtained robust approximations of marginal posterior distributions, thereby avoiding the convergence and mixing problems characteristic of traditional Bayesian methods that use Markov chain Monte Carlo sampling techniques. The analysis was conducted using the BAPC and INLA packages in R software.

The relationships between country-level age-standardized rates and MIR with the SDI were visualized through scatterplots incorporating locally weighted scatterplot smoothing regression lines and analyzed via Spearman’s correlation. The Slope Index and Concentration Index were employed to evaluate absolute and relative SDI-related health inequalities. The Slope Index was derived by regressing age-standardized rates/MIR of each age group against SDI-weighted relative positions (defined as the midpoint of cumulative population distribution ranked by SDI). The Concentration Index was calculated by integrating the area under the Lorenz curve, which illustrates the alignment between cumulative SDI-ranked population distribution and cumulative age-standardized rates/MIR distribution.

Decomposition analysis was used to clarify the individual contributions of population age structure, population growth, and epidemiological changes to appendicitis burden among children and adolescents^[14]^. By controlling for other factors and comparing ideal situations with actual outcomes, we were able to determine the extent to which specific factors influenced epidemiological trends.

Frontier analysis evaluated the potential to reduce appendicitis burden across 204 countries and territories^[14]^. This method identifies theoretical minimum age-standardized rates and MIR achievable at each country’s development level, establishing an optimal performance benchmark. The gap between observed burden and this minimum highlights improvement opportunities. Locally weighted and polynomial regression with smoothing spans (0.3, 0.4, 0.5) captured nonlinear relationships between SDI, age-standardized rates, and MIR. Robustness was ensured through 1000 bootstrap resamples, calculating average rates and MIR per SDI value. Improvement potential was assessed by comparing each country’s 2021 rates to its frontier line.

A *P*-value less than 0.05 was considered statistically significant. All analyses were performed using OriginPro 2021, GraphPad Prism 9.0, and R software (V.4.3.2).

### Ethics and STROCSS statement

Since the study utilized anonymized, publicly available epidemiological data, ethical approval was not required, and patient consent forms were unnecessary for accessing and downloading data from the database. The study followed the STROCSS guidelines for reporting cohort, cross-sectional, and case-control studies in surgery (Supplemental Digital Content 1, available at: http://links.lww.com/JS9/F155)^[15]^.

### Artificial intelligence usage statement

Our reporting adheres to the Transparency in the Reporting of Artificial Intelligence guidelines^[16]^. During manuscript preparation, the generative artificial intelligence (AI) tool DeepSeek-V3 (vendor: DeepSeek; model version: V3; accessed May 2025) was used exclusively for language polishing to improve grammar, syntax, and stylistic clarity of the authors’ original text. The tool operated with standard parameters (e.g. default temperature) via a cloud API, and no patient data or confidential materials were processed. All AI-modified content underwent rigorous author review to ensure scientific accuracy and ethical compliance. Critically, the tool was not employed for any scientific content generation, including research design, data analysis, literature synthesis, or conclusion formulation; all inputs were limited to de-identified text compliant with the General Data Protection Regulation and the Health Insurance Portability and Accountability Act, and prompts were restricted to grammatical refinement (e.g. “Improve clarity while preserving technical accuracy”). The authors confirm no conflicts of interest or financial ties with AI vendors and take full responsibility for the manuscript’s content.

## Result

### Global burden of appendicitis in children and adolescents

In 2021, there were approximately 4 534 249 new cases, 164 985 prevalent cases, 3941 deaths, and 360 249 DALYs attributed to appendicitis among children and adolescents globally (Supplemental Digital Content Table S1, available at: http://links.lww.com/JS9/F155). The age-standardized rates for incidence, prevalence, mortality, and DALYs were 165.46 (95% UI, 105.28–249.25), 6.02 (95% UI, 3.87–8.9), 0.15 (95% UI, 0.11–0.19), and 13.46 (95% UI, 10.37–17.02), respectively.

**Figure 1.**
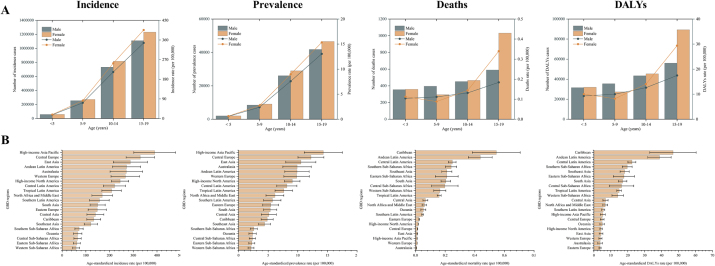
Burden of appendicitis among children and adolescents in 2021. (A) Global age-specific counts and rates of incidence, prevalence, deaths, and DALYs by sex and age groups. (B) Age-standardized incidence, prevalence, mortality, and DALY rates across 21 GBD regions in 2021.

Females exhibited a higher burden than males, with both absolute numbers and rates increasing with age, except for mortality and DALYs in the 5–9 age group (Supplemental Digital Content Table S1, available at: http://links.lww.com/JS9/F155 and Fig. 1A).

From 1990 to 2021, the age-standardized incidence and prevalence rate showed an overall slight decrease globally (incidence: AAPC, −0.06 [95% CI, −0.11 to −0.01]; prevalence: AAPC, −0.05 [95% CI, −0.10 to −0.003]) (Supplemental Digital Content Table S2, available at: http://links.lww.com/JS9/F155), but remained relatively stable among male (Supplemental Digital Content Table S3, available at: http://links.lww.com/JS9/F155 and Supplemental Digital Content Figure S1A, available at: http://links.lww.com/JS9/F155). The age-standardized mortality and DALY rates significantly declined from 1990 to 2021 (mortality: AAPC, −3.66 [95% CI, −3.94 to −3.38]); DALYs: AAPC, −3.41 [95% CI, −3.66 to −3.17]) (Supplemental Digital Content Table S2, available at: http://links.lww.com/JS9/F155), with a more pronounced decrease in males than females (Supplemental Digital Content Table S3, available at: http://links.lww.com/JS9/F155 and Supplemental Digital Content Figure S1A, available at: http://links.lww.com/JS9/F155). During the same period, age-standardized incidence and prevalence rates decreased in children under 5 years and adolescents aged 15–19 years but increased in the 5–9 and 10–14 age groups (Supplemental Digital Content Table S4, available at: http://links.lww.com/JS9/F155 and Supplemental Digital Content Figure S1B, available at: http://links.lww.com/JS9/F155). In contrast, mortality and DALY rates demonstrated a declining trend across all age groups, with a more rapid decrease observed in younger age groups.

**Figure 2.**
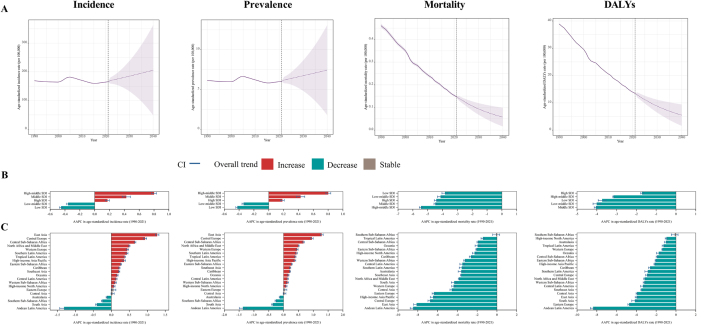
Global trends in age-standardized rates of appendicitis among children and adolescents. (A) Projected trends of age-standardized rates to 2050. (B) Temporal trends in appendicitis burden by SDI from 1990 to 2021. (C) Temporal trends in appendicitis burden by GBD regions from 1990 to 2021.

Between 2022 and 2040, the age-standardized incidence rate is expected to increase by 21.20%, from 169.73 (95% CI, 158.85–180.61) to 205.72 (95% CI, 48.62–362.82) worldwide (Fig. 2A and Supplemental Digital Content Table S5, available at: http://links.lww.com/JS9/F155). The age-standardized prevalence rate is projected to increase by 20.26%, from 6.18 (95% CI, 5.78–6.57) to 7.43 (95% CI, 1.77–13.08). Conversely, the age-standardized rates of mortality and DALYs are expected to decrease by 59.1% (from 0.14 [95% CI, 0.13–0.15] to 0.06 [95% CI, 0.02–0.1]), and 57.24% (from 12.84 [95% CI, 11.85–13.82] to 5.49 [95% CI, 1.57–9.41]), respectively (Fig. 2A and Supplemental Digital Content Table S5, available at: http://links.lww.com/JS9/F155).

**Figure 3.**
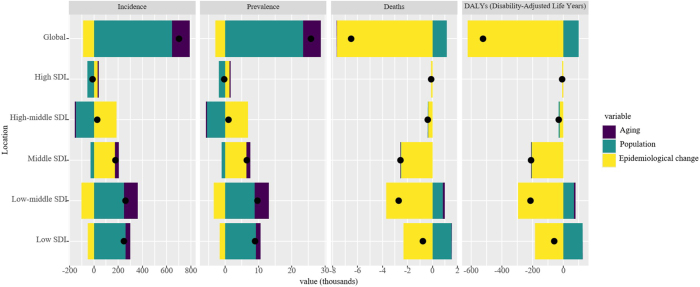
Decomposition analysis of the trends in children and adolescents’ appendicitis incidence, prevalence, deaths, and DALYs from 1990 to 2021.

By performing decomposition analysis, we evaluated the impact of aging, population growth, and epidemiological changes on appendicitis epidemiology from 1990 to 2021 (Fig. 3 and Supplemental Digital Content Table S6, available at: http://links.lww.com/JS9/F155). Globally, incidence and prevalence showed upward trends, driven primarily by population growth (incidence: 91.88%; prevalence: 90.9%), with smaller contributions from aging (incidence: 20.74%; prevalence: 20.54%) and offset by negative epidemiological changes (incidence: −12.61%; prevalence: −11.44%). In contrast, deaths and DALYs exhibited downward trends, largely due to epidemiological improvements (deaths: 117.64%; DALYs: 118.87%), while population growth had a negative impact (deaths: −17.7%; DALYs: −19.01%) and aging contributed minimally (deaths: 0.06%; DALYs: 0.14%).

### Appendicitis in children and adolescents among different SDI regions

In 2021, an obvious stepwise gradient in age-standardized incidence and prevalence was observed across SDI levels, with higher rates corresponding to increasing SDI.

The high SDI recorded the highest incidence and prevalence rate, while the low SDI had the lowest rates (Supplemental Digital Content Table S1, available at: http://links.lww.com/JS9/F155). Conversely, mortality and DALY rates demonstrated a declining trend with increasing SDI levels. The low SDI exhibited the highest mortality and DALYs, whereas the high SDI had the lowest rates.

**Figure 4.**
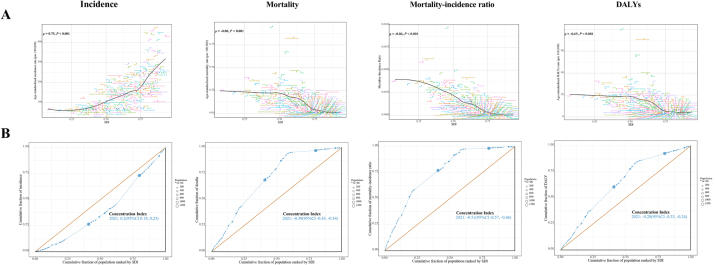
SDI-related inequalities in appendicitis burden among children and adolescents, 2021. (A) Associations between incidence rate, mortality rate, mortality-incidence-ratio, and DALY rates with SDI. (B) Concentration curve illustrating SDI-related inequalities in age-standardized rates and incidence-mortality ratio.

Pearson correlation analysis revealed incidence was positively correlated with SDI at 204 countries (*R* = 0.75), while mortality (*R* = −0.80), MIR (*R* = −0.86), and DALYs (*R* = −0.69) were negatively correlated with SDI (Fig. 4A). Comprehensive measures monitoring SDI-related inequalities in the burden of appendicitis are shown in Figure 4B and Supplemental Digital Content Table S7, available at: http://links.lww.com/JS9/F155. These findings underscore significant absolute and relative health inequalities: higher SDI countries experience disproportionately higher incidence, while lower SDI countries bear a disproportionately higher burden of mortality, MIR, and DALYs.

From 1990 to 2021, incidence and prevalence rates increased significantly in middle to high SDI, with the most substantial rise in the high-middle SDI. In contrast, low and low-middle SDI experienced significant declines, particularly in the low SDI (Fig. 2B, Supplemental Digital Content Figure S1C, available at: http://links.lww.com/JS9/F155, and Supplemental Digital Content Table S2, available at: http://links.lww.com/JS9/F155). Additionally, mortality and DALY rates declined across all SDI levels, with the steepest reductions in high-middle SDI regions.

Decomposition analysis revealed that, except for high SDI regions, incidence and prevalence increased across all SDI regions, with the most substantial rise observed in low-middle SDI regions (Fig. 3 and Supplemental Digital Content Table S6, available at: http://links.lww.com/JS9/F155). Population growth showed varying impacts, ranging from −544.80% to 475.36% for incidence and −564.26% to 613.55% for prevalence across regions. Aging contributed positively in low-middle SDI regions, but negatively in high SDI regions. Epidemiological changes were significant in high-middle SDI regions. Deaths and DALYs declined across all SDI regions, with the most substantial reductions in low-middle SDI regions, driven largely by epidemiological improvements, particularly in low SDI regions.

### Appendicitis in children and adolescents among 21 GBD regions

In 2021, high-income Asia Pacific exhibited the highest incidence and prevalence rates, while Western Sub-Saharan Africa had the lowest incidence and prevalence rates (Supplemental Digital Content Table S1, available at: http://links.lww.com/JS9/F155 and Fig. 1B). Meanwhile, the highest mortality and DALY rates were observed in the Caribbean.

From 1990 to 2021, 15 of 21 GBD regions showed increasing trends in age-standardized incidence and prevalence rates, with the highest increase in East Asia (Fig. 2C and Supplemental Digital Content Table S2, available at: http://links.lww.com/JS9/F155). Only four regions exhibited decreasing trends, led by Andean Latin America. Mortality and DALY rates declined across all 21 regions, with the most significant reductions in Andean Latin America.

Between 2022 and 2040, the age-standardized incidence rate of appendicitis is projected to increase in all regions except high-income Asia Pacific and South Asia (−3.42%), with the largest rise in South Asia (51.25%) (Supplemental Digital Content Table S5, available at: http://links.lww.com/JS9/F155 and Supplemental Digital Content Figure S2, available at: http://links.lww.com/JS9/F155). Similarly, the prevalence rate is expected to rise in all regions except high-income Asia Pacific and Western Europe, with the most significant increase in South Asia (47.91%). Age-standardized mortality and DALY rates are predicted to decline across all regions except Southern Sub-Saharan Africa (mortality: 30.80%; DALYs: 23.83%), with the steepest reductions in Australasia (−100%).

### Appendicitis in children and adolescents among 204 countries and territories

**Figure 5.**
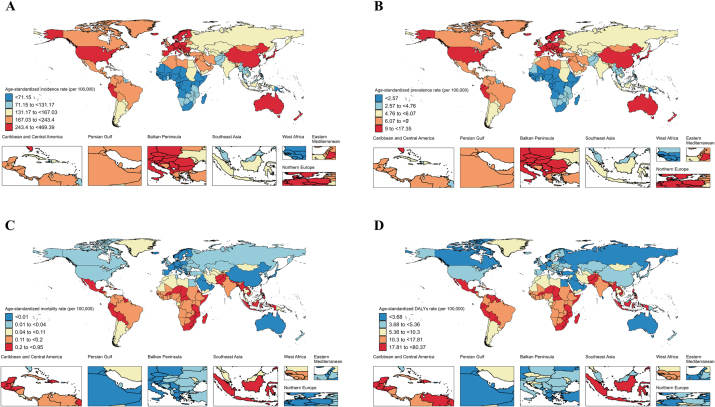
Age-standardized incidence (A), prevalence (B), mortality (C), and DALYs (D) rates of appendicitis among children and adolescents in 2021, by country and territory.

In 2021, among 204 countries and territories, Belgium had the highest age-standardized incidence and prevalence rate, while China recorded the highest number of new cases and prevalent cases (Fig. 5A–D and Supplemental Digital Content Table S8, available at: http://links.lww.com/JS9/F155). Conversely, Ethiopia recorded the incidence and prevalence rates, and Tokelau reported the fewest new and prevalent cases. Haiti exhibited the highest mortality and DALYs, while India recorded the highest number of deaths and DALYs.

From 1990 to 2021, Czechia experienced the largest increase in age-standardized incidence and prevalence (Supplemental Digital Content Figure S3A–D, available at: http://links.lww.com/JS9/F155 and Supplemental Digital Content Table S9, available at: http://links.lww.com/JS9/F155). In contrast, Guatemala saw the steepest declines, with an AAPC of −1.83 (95% CI: −1.98 to −1.47) for incidence and −1.87 (95% CI: −2.02 to −1.72) for prevalence. Among 204 countries and territories, only Niue and Tokelau showed increases in mortality rates, while Niue, Tokelau, and Zimbabwe experienced rises in DALY rates. Peru recorded the largest reductions in mortality and DALY rates.

**Figure 6.**
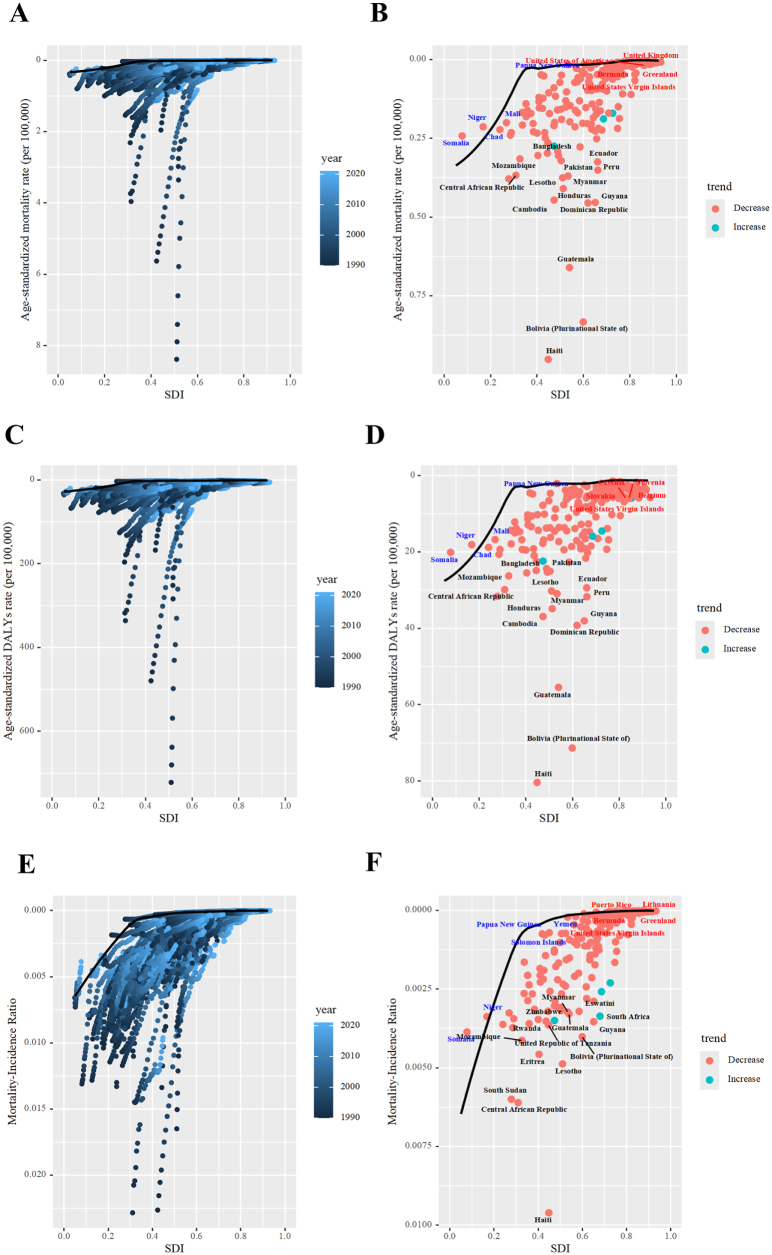
Frontier analysis exploring the relationship between mortality (A, B), DALYs (C, D), mortality-incidence-ratio (E, F), and SDI for appendicitis among children and adolescents in 204 countries and territories.

Frontier analysis indicated that, for age-standardized mortality and DALY rate, Haiti, Bolivia, and Guatemala showed the largest gaps between current performance and potential improvement (Fig. 6A and B and Supplemental Digital Content Tables S10 and S11, available at: http://links.lww.com/JS9/F155). Low-SDI frontier countries included Somalia, Niger, Chad, Mali, and Papua New Guinea. Among high-SDI regions, the United States Virgin Islands, Greenland, Bermuda, the United States, and the United Kingdom had notable potential for reducing mortality rates, while Slovakia, Belgium, Czechia, and Slovenia showed significant potential for DALYs improvement. For MIR, the largest improvement gaps were observed in Haiti, the Central African Republic, South Sudan, and Lesotho (Fig. 6C and Supplemental Digital Content Table S12, available at: http://links.lww.com/JS9/F155). Low-SDI frontier countries for MIR included Somalia, Niger, Solomon Islands, Papua New Guinea, and Yemen, while high-SDI regions with improvement potential included the United States Virgin Islands, Bermuda, Greenland, Puerto Rico, and Lithuania.

## Discussion

To our knowledge, this is the first comprehensive global, regional, and national study on appendicitis burden among children and adolescents aged 0–19 years. Our findings reveal significant disparities in incidence, prevalence, mortality, and DALYs across socioeconomic development levels and regions, with notable temporal and demographic variations. Low SDI regions bear disproportionately high mortality and DALYs burdens, while high SDI regions face higher incidence and prevalence. Although global mortality and DALY rates declined from 1990 to 2021, incidence and prevalence rates have risen annually since 2015 and are projected to increase by over 20%. These results highlight a persistent appendicitis burden in this vulnerable population, emphasizing the urgent need for tailored interventions.

This study found that age-standardized incidence and prevalence rates of appendicitis in children and adolescents are positively correlated with SDI levels, while mortality and DALYs show a negative correlation. These findings align with studies across all age groups^[2,8]^, which report higher incidence and prevalence in high-SDI regions but greater mortality and DALYs in low-SDI regions. Advanced diagnostic tools, such as ultrasound and CT, contribute to higher detection rates in high-SDI regions, including subclinical cases^[17]^. Dietary patterns low in fiber and high in processed foods may elevate appendicitis risk through multiple pathways: (1) Reduced dietary fiber intake diminishes fecal bulk and prolongs intestinal transit time, thereby increasing the risk of luminal obstruction in the appendix^[8]^; (2) processed foods disrupt gut microbiota composition (e.g. reducing Bacteroidetes diversity), which may trigger mucosal inflammation and lymphoid hyperplasia in the appendiceal wall^[18–20]^; (3) urbanization and sedentary lifestyles further exacerbate these effects by impairing circadian rhythms and immune homeostasis^[21]^. Additionally, excessive hygiene and early use of antibiotics in high-SDI regions may alter the gut microbiota, theoretically influencing immune regulation and appendiceal lymphoid tissue reactivity. However, the mechanistic contributions of these factors to appendicitis pathogenesis remain poorly understood and controversial. At the same time, obesity is a risk factor for multiple diseases, including appendicitis, and obesity rates are often higher in high-SDI regions^[22]^. In high-SDI regions, reducing diagnostic overuse and resource waste is crucial, with adherence to evidence-based guidelines and refined diagnostic algorithms minimizing unnecessary interventions. Concurrently implementation of antibiotic stewardship programs for uncomplicated cases and advance quality improvement initiatives for surgical outcomes. Although non-operative management (antibiotic therapy alone) for uncomplicated appendicitis has garnered increasing attention^[23,24]^, we maintain a cautious stance toward its universal adoption. Recurrent inflammation within weeks to months may precipitate adhesion formation, thereby increasing the technical complexity of subsequent interventions and elevating perioperative risks. Moreover, in cases where abdominal pain lacks classic acute abdomen features, clinicians should rigorously exclude inaugural presentations of inflammatory bowel disease – a critical differential diagnosis necessitating distinct therapeutic strategies. Addressing underlying risk factors may help reduce incidence. Conversely, the observed lower incidence of appendicitis in low-SDI regions likely stems from both protective biological factors and systemic surveillance gaps, though this apparent advantage is overshadowed by catastrophic outcome disparities. Traditional diets rich in dietary fiber may confer genuine protection against luminal obstruction, while epidemiological transition delays the adoption of Westernized risk factors like obesity. However, profound underdiagnosis – driven by limited healthcare access, inadequate imaging resources, and community-level data deficiencies – artificially suppresses reported rates. Paradoxically, these populations suffer disproportionately high mortality and DALYs, revealing how structural healthcare barriers convert a routinely manageable condition into life-threatening emergencies. This phenomenon redefines the observed “low incidence” as an artifact of surveillance limitations rather than a true epidemiological advantage. In low-SDI regions, higher mortality and DALYs may stem from constrained surgical care, suboptimal diagnostic capacity, healthcare resource shortages, and systemic barriers, where delayed treatment and elevated perforation rates further exacerbate poor outcomes^[25,26]^. Investments in infrastructure, healthcare provider training, emergency care, and early detection are essential. Innovative strategies, such as task-shifting to nonphysician providers for basic surgeries and international collaboration to mobilize resources, can help reduce global healthcare inequalities^[27]^. In addition, community health workers can be equipped with point-of-care ultrasound devices and simplified scoring systems (Alvarado score ≥7) for rapid triage and referral, drawing on the successful community diagnostic model implemented in Papua New Guinea.

From 1990 to 2021, age-standardized incidence and prevalence rates of appendicitis showed modest declines globally, while absolute case numbers rose due to population growth and demographic changes. These trends underscore the dual impact of healthcare improvements and demographic transitions. Mortality and DALYs have declined substantially, reflecting advances in surgical techniques, anesthesia, perioperative care, and the adoption of evidence-based management strategies, such as non-operative management for uncomplicated appendicitis, particularly in high-SDI regions^[28]^. However, these advancements remain unevenly distributed across regions. These improvements reflect broader trends in global health, where attention and investment from many countries have led to continued reductions in health loss among children and adolescents^[29]^. At the same time, the epidemiological focus has progressively shifted toward injuries and non-communicable diseases^[29]^. From 2022 to 2040, the projected rise in incidence and prevalence reflects ongoing changes in healthcare access, population structure, and risk factor profiles^[8]^. Based on the above results, it is reasonable to infer that the governance of appendicitis is urgent. This forecast data can provide evidence-based support for long-term planning. In contrast, the continued decline in mortality and DALY rates is encouraging, suggesting that global health initiatives and surgical advancements are effectively reducing the most severe outcomes of appendicitis.

The burden of appendicitis increases with age, with adolescents aged 15–19 years experiencing the highest incidence and prevalence rates. This trend may be linked to the immunological and physiological changes associated with puberty, such as increased lymphoid follicle hyperplasia in the appendix, which predisposes this age group to luminal obstruction and subsequent inflammation^[30]^. Notably, although age-standardized mortality and DALY rates generally increase with age among children and adolescents, the age-standardized mortality rate in children under 5 years is higher than that in the 5–9-year age group. This exception may be attributed to the diagnostic challenges associated with atypical clinical presentations and communication barriers in children under 5 years, which often lead to delayed diagnosis, higher rates of perforation, and poorer clinical outcomes^[30]^. Efforts to improve early detection in this vulnerable group should include education for caregivers and the use of advanced diagnostic tools. Notably, while our 5-year age stratification aligns with global comparative studies^[22,31,32]^, we recognize that even within these narrower age brackets (e.g. between 15- and 19-year-olds), significant differences undoubtedly exist. Future studies may benefit from further age subgroup analyses to explore these developmental variations. As with the results for all age groups previously reported^[33]^, global female children and adolescents have a slightly higher burden of appendicitis, particularly in the older age groups. However, this gender difference varies across regions, and no statistically significant difference in burden was observed between males and females. Therefore, in prevention and treatment policies, equal attention should be given to both females and males.

This study used frontier analysis to assess the potential for improving appendicitis outcomes in children and adolescents across 204 countries, considering age-standardized mortality rates, DALYs, MIR, and SDI. Unlike traditional regression models, frontier analysis identifies the theoretical minimum burden of appendicitis at a given socio-economic development level^[14]^. This method highlights underperforming regions by quantifying the gap between current outcomes and optimal performance, providing insights for targeted interventions. Low-SDI countries like Somalia, Niger, and Papua New Guinea emerged as frontier regions, demonstrating strong performance in reducing appendicitis-related mortality, DALYs, and MIR despite limited resources. These achievements may be due to resource-efficient strategies, such as task-shifting and community health initiatives, which have improved surgical outcomes and reduced diagnostic delays^[34,35]^. The policies in these regions should be further explored to inform strategies for other resource-limited areas. Conversely, some high-SDI regions, like the United States Virgin Islands, showed potential for improvement, indicating inefficiencies in healthcare delivery or disparities that limit outcomes. Despite having advanced diagnostic and surgical capabilities, these regions must focus on optimizing care pathways, addressing inequities, and ensuring timely access for all populations. For countries with large gaps from the frontier burden, efforts to manage appendicitis should be intensified, regardless of SDI. These findings highlight the need for tailored health policies and interventions. By learning from frontier regions and addressing inefficiencies in others, countries can reduce the global burden of appendicitis in children and adolescents and improve health equity.

Our study had limitations. First, estimates in regions with scarce primary data rely heavily on statistical modeling, highlighting the need for more comprehensive and reliable data collection to improve accuracy^[31]^. Second, misdiagnosis and underdiagnosis in resource-limited areas with weak healthcare systems may lead to underestimation of disease burden^[31]^. Finally, the GBD database aggregates all appendicitis types without distinguishing perforated from non-perforated cases, limiting subtype-specific burden and outcome assessments^[36]^.

## Conclusions

This study underscores the evolving global burden of appendicitis in children and adolescents, shaped by demographic, socioeconomic, and healthcare factors. Addressing these disparities demands coordinated efforts to improve surgical access, enhance prevention, and ensure equitable healthcare. By investing in health systems and expanding appendicitis research, we can mitigate the burden of this common yet life-threatening condition. Focusing on children and adolescents provides new insights into their disease burden, highlighting the need for age- and region-specific strategies to tackle the growing global challenge of appendicitis.

## Data Availability

The original data used in the present study can be accessed at the 2021 Global Burden of Disease (GBD) study (https://vizhub.healthdata.org/gbd-results/). The analyzed datasets during the current study are available from the corresponding author on reasonable request.
